# The Resource Utilization of Poplar Leaves for CO_2_ Adsorption

**DOI:** 10.3390/molecules29092024

**Published:** 2024-04-27

**Authors:** Xia Wang, Fanyuan Kong, Wulan Zeng, Huaxiang Zhang, Chunling Xin, Xiangjun Kong

**Affiliations:** 1 Department of Chemistry and Chemical Engineering, Weifang University, Weifang 261061, China; 2 Library, Weifang University, Weifang 261061, China

**Keywords:** activated carbons, activation, nitrogen doping, CO_2_ adsorption, regeneration

## Abstract

Every late autumn, fluttering poplar leaves scatter throughout the campus and city streets. In this work, poplar leaves were used as the raw material, while H_3_PO_4_ and KOH were used as activators and urea was used as the nitrogen source to prepare biomass based-activated carbons (ACs) to capture CO_2_. The pore structures, functional groups and morphology, and desorption performance of the prepared ACs were characterized; the CO_2_ adsorption, regeneration, and kinetics were also evaluated. The results showed that H_3_PO_4_ and urea obviously promoted the development of pore structures and pyrrole nitrogen (N–5), while KOH and urea were more conductive to the formation of hydroxyl (–OH) and ether (C–O) functional groups. At optimal operating conditions, the CO_2_ adsorption capacity of H_3_PO_4_– and KOH–activated poplar leaves after urea treatment reached 4.07 and 3.85 mmol/g, respectively, at room temperature; both showed stable regenerative behaviour after ten adsorption–desorption cycles.

## 1. Introduction

Currently, excessive CO_2_ emissions cause serious greenhouse gas (GHG) effects and increase the burden of achieving dual carbon targets. Liquid absorption and solid adsorption methods have been widely studied for separating CO_2_ from large CO_2_ emission points [1,2,3,4]. To date, liquid absorption technology has been applied in industry, but the most commonly used liquid amines have the disadvantages of easy degradation, high regeneration energy consumption, and strong corrosion at equilibrium [5,6,7,8,9]. The solid adsorption method is well approved due to its good adsorption capacity, low corrosiveness, and low degradation [10,11]. Solid sorbents, such as molecular sieves [9,12,13], metal-organic frameworks (MOFs) [14,15,16,17], carbon nanotubes [18], microporous carbons [19,20,21,22,23], and other synthetic and modified materials [24,25,26,27,28,29,30,31,32,33,34], exhibit good CO_2_ adsorption performance. However, the synthesis costs of these sorbents are usually high, which undoubtedly hinders their wide application.

Realizing effective CO_2_ capture at large CO_2_ emission points at low costs is a significant contribution. In late autumn in China, large amounts of waste biomass, such as agricultural waste and leaves, are stacked in disorderly fashion and burned traditionally, causing serious environmental pollution problems and hindering dual carbon targets. The resource utilization of biomass waste is undoubtedly a win-win situation.

Many researchers have conducted extensive studies on realizing the resource utilization of biomass as a carrier of catalyst or an adsorbent for gas and heavy metals [35,36,37,38,39,40,41,42,43,44,45,46,47,48,49,50,51,52,53,54]. Zhang et al. designed poly(acrylic acid)–grafted chitosan and rice straw-based biochar to adsorb heavy metals in wastewater, which suggested selective adsorption of Cr^3+^, Pb^2+^, and Cu^2+^ [36]. Ding et al. prepared seaweed-based porous biochars from Sargassum and Enteromorpha using a KOH activation method, and CO_2_ adsorption capacities of 1.05 and 0.52 mmol/g were reached for both at room temperature [37]. Xu et al. prepared N-doped biochars from waste walnut shells by using urea as the nitrogen source, H_3_PO_4_ as the pretreatment agent and KOH, K_2_CO_3_, and ZnCl_2_ as activators; the results showed that different activators suggested different effects on improving the pore structure and N content. The CO_2_ adsorption capacity of ZnCl_2_-activated waste walnut shells was 0.6 mmol/g at 25 °C and 0.15 bar [41]. Ello et al. prepared microporous biocarbon materials from African palm shells using a KOH-activation method; the specific surface area ranged from 365 to 1890 m^2^/g, the pore volume ranged from 0.16 to 0.82 cm^3^/g, and the CO_2_ adsorption capacity was 4.4 mmol/g at 25 °C and 1 bar [43]. Plaza et al. produced microporous biochars from almond shells using one-step activation with an O_2_ concentration of 3–5% in a N_2_ atmosphere at 500–650 °C, and the results showed that the developed narrow microporosity promoted CO_2_ adsorption at low partial pressures [47]. Our research group also prepared corncob- and peanut-shell-based activated carbons using an alkali activation method, which exhibited developed pore structures and good CO_2_ adsorption performance [50,51].

Poplars are among the most common green tree species in China, and poplar leaves also scatter on roads in late autumn; therefore, understanding the resource utilization of poplar leaves has great practical significance. Here, fallen and yellow poplar leaves were used as the raw material, H_3_PO_4_ and KOH were used as activators, and urea was used as the nitrogen source to prepare ACs with abundant surface functional groups and pore structures. The pore structures and surface morphology, types and ratios of surface nitrogeN- and oxygen-containing groups, and desorption features of the activated poplar leaves before and after urea treatment were characterized, and the adsorption and regeneration performance were also tested.

## 2. Results and Discussion

### 2.1. Characterization

**Figure 1:**
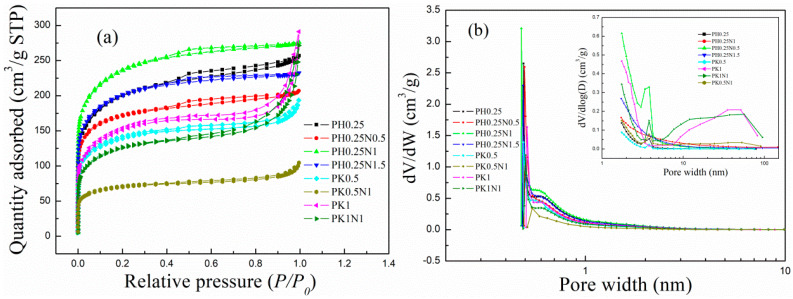
The (**a**) N_2_ adsorption–desorption isotherms, and (**b**) pore size distribution curves of H_3_PO_4_– or KOH–activated poplar leaves before and after N-doping.

(1) N_2_ adsorption–desorption. The N_2_ adsorption–desorption isotherms and pore size distribution curves for the H_3_PO_4_– and KOH–activated poplar leaves before and after N-doping are shown in Figure 1a,b, respectively. As shown in Figure 1a, in the initial stage of increasing partial pressure, the N_2_ adsorption of the characterized ACs sharply increased, which is an indication of the existence of a large number of micropores [20,55]; as the relative pressure continuously increased, the N_2_ adsorption slowly increased, and hysteresis loops appeared in the final stage, suggesting that mesopores also appeared in the activated poplar leaves [26,27]. In other words, both micropores and mesopores appeared in the poplar leaf-based ACs. From the pore size distribution curves in Figure 1b, the micropore size is mainly less than 1 nm, which is favourable for the physical adsorption of CO_2_ [19,43]. The textural pore properties of the activated poplar leaves are shown in Table S1. As suggested in Table S1, the activated poplar leaves before and after N-doping had large specific surface areas and pore volumes ranging from 416–846 m^2^/g and 0.16–0.45 cm^3^/g, respectively. For H_3_PO_4_–activated poplar leaves, especially PH0.25N1, the pore properties, such as the specific surface area and pore volume, exhibited greater advantages than did those of KOH-activated poplar leaves.

(2) XPS spectrum. Based on previous research findings, the hydroxyl functional group of –OH, the ether functional groups of C–O and the pyrrolic nitrogen functional group of N–5 are favourable for CO_2_ adsorption [41,50], so the O and N species, especially the ratios of –OH, C–O and N–5 in the prepared ACs, have received increased attention.

**Figure 2:**
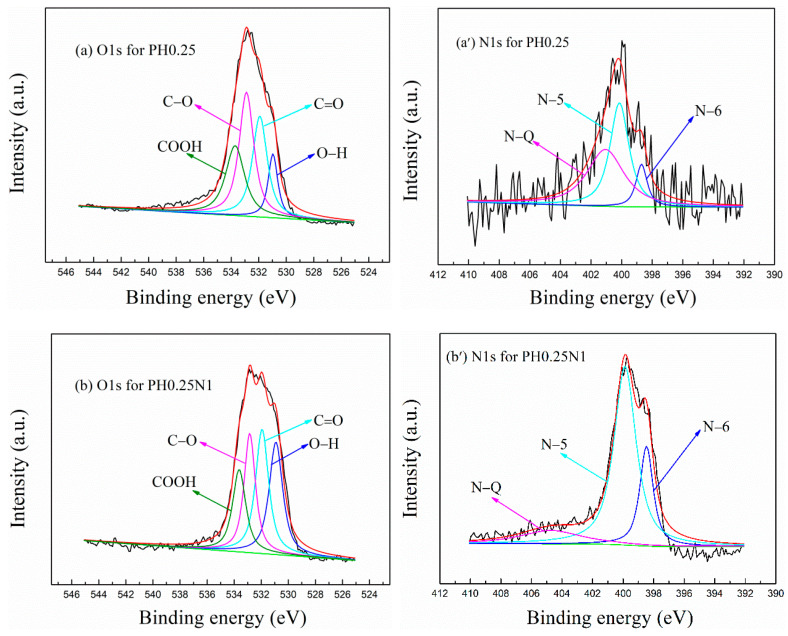

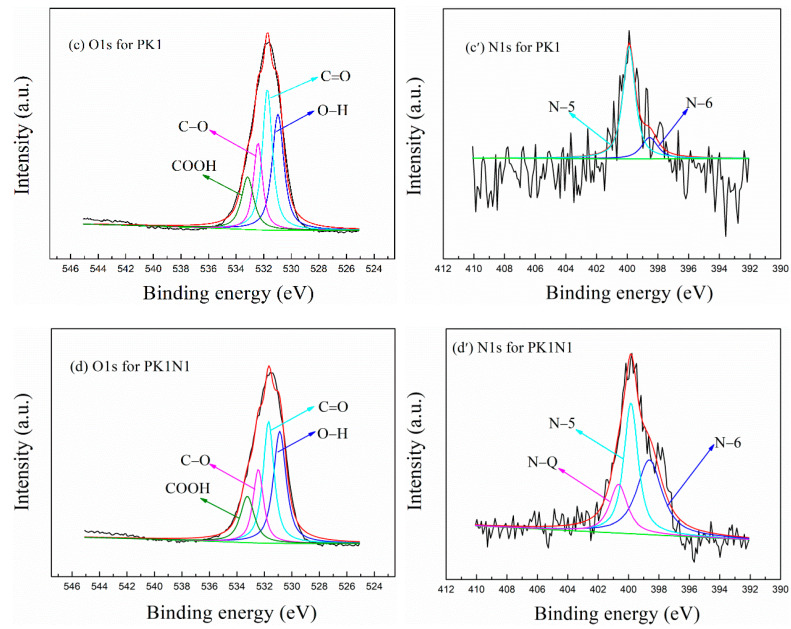
XPS spectra of H_3_PO_4_– or KOH–activated poplar leaves before and after N-doping. (**a**–**d**): O1S, (**a′**–**d′**): N1S.

The XPS spectra for O1S and N1S of the H_3_PO_4_– and KOH-activated poplar leaves before and after N-doping are shown in Figure 2a–d and Figure 2a′–d′, respectively, and the peak area ratios of the O and N species are shown in Table S2 and Table S3, respectively. As shown in Figure 2a–d and Table S2, the total ratios of –OH and C–O for PH0.25 and PH0.25N1 are 11.04% and 10.21%, respectively, and the corresponding values for PK1 and PK1N1 are 24.47% and 18.74%, respectively; N-doping slightly reduces the amount of oxygen-containing functional groups, which may be caused by competition and interaction with nitrogen-containing functional groups. By comparison, the KOH–activated poplar leaves exhibited greater advantages in terms of oxygen-containing functional groups than the H_3_PO_4_–activated poplar leaves.

As shown in Figure 2a′–d′ and Table S3, the ratios of N–5 for PH0.25 and PH0.25N1 are 0.96% and 6.38%, and the corresponding values for PK1 and PK1N1 are 2.09% and 1.58%, respectively. N-doping significantly increased the amount of nitrogen-containing functional groups on the H_3_PO_4_–activated poplar leaves, but slightly reduced the amount of KOH–activated poplar leaves, which may be caused by the strong corrosiveness of KOH or the competitive formation of quaternary nitrogen functional groups (N–Q).

(3) SEM

**Figure 3:**
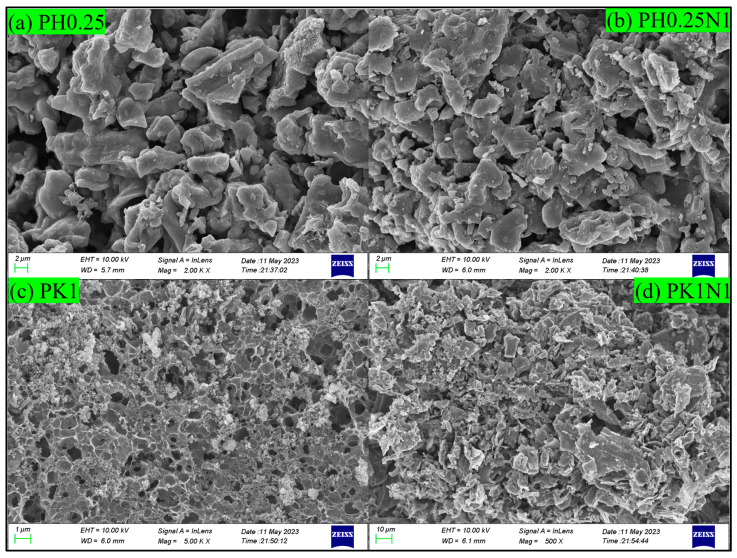
SEM images of H_3_PO_4_– or KOH–activated poplar leaves before and after N-doping.

The surface morphology images of the H_3_PO_4_– and KOH–activated poplar leaves before and after N-doping are shown in Figure 3a–d. As shown in the Figures, the activation and N-doping of the poplar leaves led to the formation of developed and irregular pores. As shown in Figure 3a,b, the pores in PH0.25 are coarse and more developed and layered. As shown in Figure 3c,d, the KOH-activated poplar leaves formed dense and nearly circular pores, and after further N-doping, the pore surfaces were destroyed and became more disordered. By comparison, the surface morphology of the H_3_PO_4_–activated poplar leaves before and after N-doping was more suitable for CO_2_ adsorption, and the characterization results were consistent with those of the BET characterization. The differences in the pore structure and surface morphology between H_3_PO_4_ and KOH activation were mainly attributed to the activation mechanism and corrosiveness of H_3_PO_4_ and KOH.

(4) CO_2_-TPD

**Figure 4:**
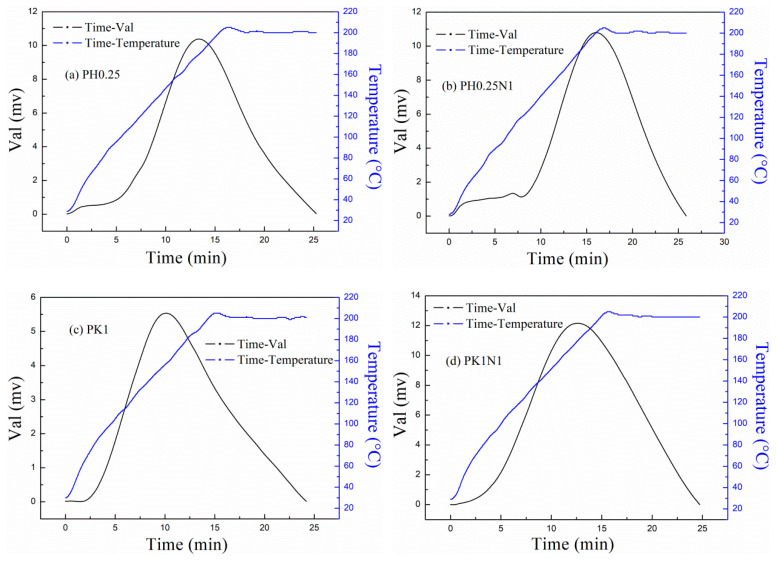
CO_2_-TPD curves of H_3_PO_4_– or KOH–activated poplar leaves before and after N-doping.

The CO_2_-TPD curves for the H_3_PO_4_– and KOH–activated poplar leaves before and after N-doping are displayed in Figure 4a–d, which demonstrate the CO_2_ desorption condition, and the initial adsorption temperature of the samples was set to 20 °C. As shown in Figure 4, as the temperature increased, the desorption phenomenon gradually appeared and became obvious at relatively low temperatures, suggesting that the adsorption process was mainly based on physisorption. However, the curve peaks for the studied sorbents all appeared at nearly 200 °C, which indicates that chemisorption was also involved in the adsorption process. The physisorption was mainly due to the narrow micropores and the hydrogen-bonding interactions between CO_2_ and the O- or N-containing functional groups, and the chemisorption was mainly due to the reaction between the pyrrolic nitrogen functional groups and CO_2_.

For the N-doped sorbents, the desorption peaks appeared later than those of the H_3_PO_4_– and KOH–activated poplar leaves, which was mainly caused by their stronger adsorption capacity and greater chemisorption.

### 2.2. CO_2_ Adsorption Properties of Poplar Leaves-Based ACs

#### 2.2.1. CO_2_ Adsorption Performance and Process Optimization

**Figure 5:**
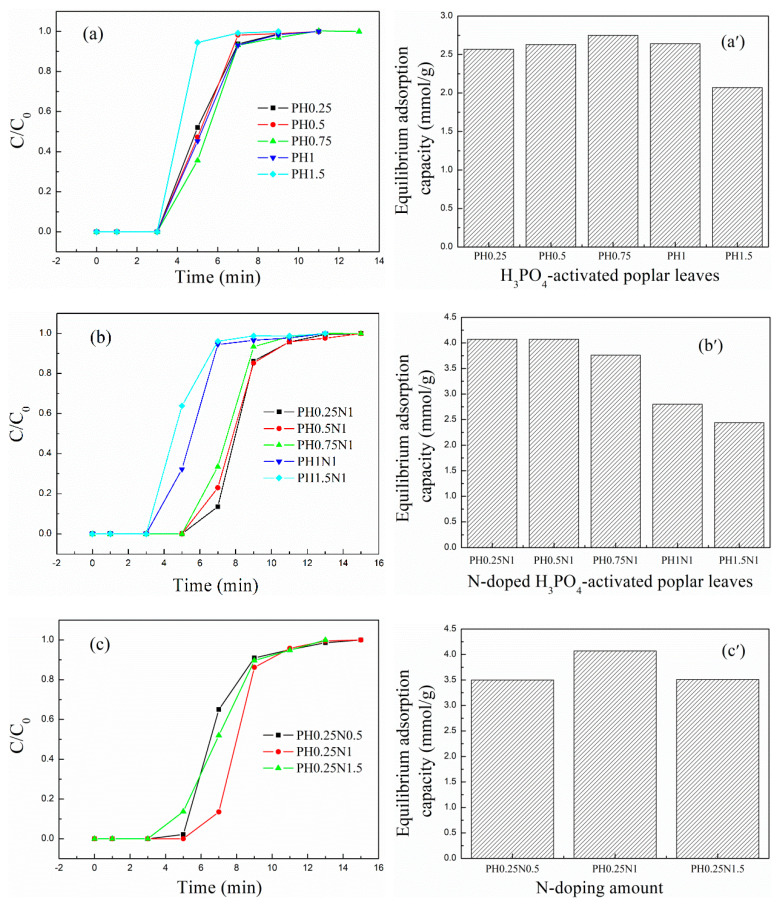

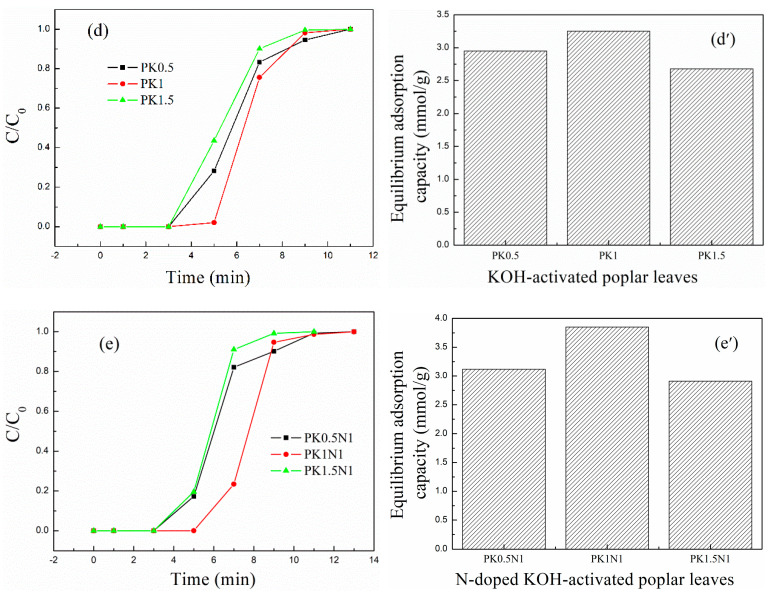
(**a**–**e**) CO_2_ breakthrough adsorption curves and (**a**′–**e**′) equilibrium adsorption capacities of H_3_PO_4_– or KOH–activated poplar leaves before and after N-doping.

Considering the factors that may affect the formation of pores and functional groups, the CO_2_ adsorption performance of the ACs prepared with different volume ratios of H_3_PO_4_ to poplar leaves, mass ratios of KOH to poplar leaves, mass ratios of urea to H_3_PO_4_, and KOH–activated poplar leaves were investigated, and the breakthrough adsorption curves and equilibrium adsorption capacities are shown in Figure 5a–e and Figure 5a′–e′, respectively.

As shown in Figure 5a,b,a′,b′, as the volume ratio of H_3_PO_4_ to poplar leaves increased from 0.25 to 1, the equilibrium adsorption capacity of the H_3_PO_4_–activated poplar leaves ranged between 2.57 and 2.74 mmol/g, which did not change much. However, as the ratio further increased to 1.5, the adsorption capacity decreased to 2.07 mmol/g. After further N-doping, with a 1:1 mass ratio of urea to H_3_PO_4_-activated poplar leaves, the CO_2_ adsorption capacity increased significantly compared with that of the H_3_PO_4_–activated poplar leaves before N-doping; the CO_2_ adsorption capacity ranged between 2.44 and 4.07 mmol/g, and exhibited a decreasing trend as the volume ratio of H_3_PO_4_ to poplar leaves increased. Considering both the preparation cost and the CO_2_ adsorption capacity, the volume ratio of H_3_PO_4_ to poplar leaves was determined to be 0.25 to further investigate the effect of the N dopant amount.

The breakthrough adsorption curves and CO_2_ adsorption capacities for different mass ratios of urea to PH0.25 are shown in Figure 5c and Figure 5c′, respectively. As the mass ratio increased from 0.5 to 1.5, the CO_2_ adsorption performance first increased and then decreased, with a value of 4.07 mmol/g for the maximum PH0.25N1. In summary, when the volume ratio of H_3_PO_4_ to poplar leaves was 0.25:1 and the activation temperature was 450 °C, and the mass ratio of urea to H_3_PO_4_-activated poplar leaves was 1:1 and the calcination temperature was 350 °C, the CO_2_ adsorption performance for PH0.25N1 reached 4.07 mmol/g at 20 °C, which was comparable to the results of relative studies [41,42,43,44,49,50,51,52].

For KOH– and urea-modified poplar leaves, the adsorption performances are shown in Figure 5d,e and Figure 5d′,e′, respectively, and the N-doping treatment also increased the adsorption capacity of the KOH-activated poplar leaves. When the mass ratio of KOH to poplar leaves was 1:1 and the activation temperature was 700 °C, and the mass ratio of urea to PK1 was 1:1 and the calcination temperature was 350 °C, the CO_2_ adsorption performance for PK1N1 reached 3.85 mmol/g at 20 °C, which was relatively lower than that of PH0.25N1.

In summary, from the view of material cost, corrosiveness, process energy consumption, and adsorption performance, H_3_PO_4_ has more advantages than KOH as an activator of poplar leaves.

#### 2.2.2. The Adsorption Kinetics of the Poplar Leaf-Based ACs

**Figure 6:**
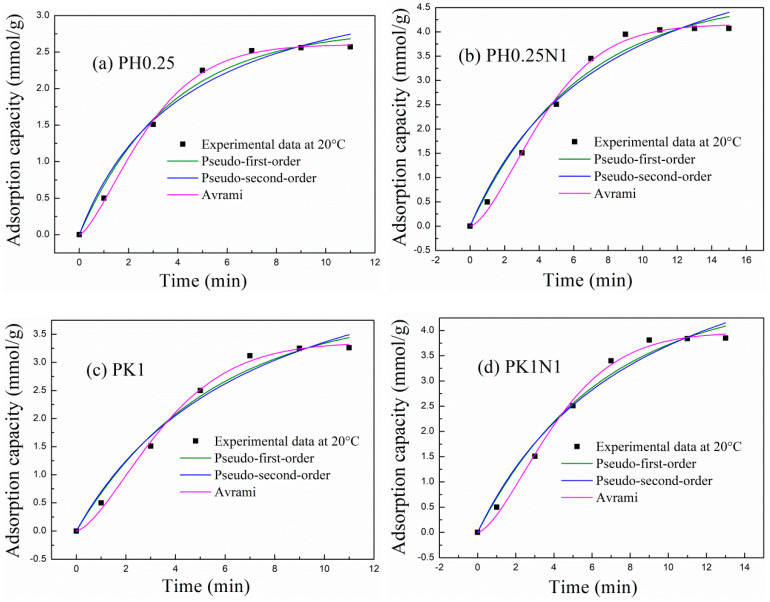
Nonlinear fitting curves of the experimental adsorption data of H_3_PO_4_– or KOH–activated poplar leaves before and after N-doping according to different kinetic models.

Adsorption kinetics are also important indices for evaluating the adsorption properties of an adsorbent. The pseudo-first-order, pseudo-second-order and Avrami models were used to simulate the adsorption kinetics of H_3_PO_4_– and KOH–activated poplar leaves before and after N-doping. The fitting curves are shown in Figure 6, and the fitting parameters are shown in Table 1. From the fitting results, only the Avrami model was well fitted to the experimental adsorption capacities of PH0.25, PH0.25N1, PK1, and PK1N1. The variance in R^2^ ranged between 0.9948 and 0.9982, and n_a_ ranged between 1.4272 and 1.5571, suggesting that the Avrami model is more suitable for describing the adsorption kinetics of poplar leaf-based ACs than the pseudo-first-order and pseudo-second-order models. Adsorption involves not only physisorption or chemisorption, but also a comprehensive adsorption process [27,28,33].

For PH0.25 and PH0.25N1, the adsorption rate constants are 0.3146 and 0.2016 min^−1^, respectively, while they are 0.2485 and 0.2108 min^−1^ for PK1 and PK1N1, respectively. For the H_3_PO_4_– and KOH–activated poplar leaves after N-doping, the adsorption rate constants all decreased, which corresponded with decreased adsorption rates. Combined with the above mentioned XPS characterization, these findings indicate that N-doping results in more N- and O-containing functional groups, which promote more chemisorption during the adsorption process, and that the adsorption rate decreases.

The adsorption data were further treated with the intraparticle diffusion model, and the fitting results are shown in Figure S1. From the fitting results, the third adsorption stage had the smallest slope of 0.14, suggesting that the equilibrium adsorption stage was the rate-controlling step, and the boundary layer diffusion and intraparticle diffusion stages were quick.

#### 2.2.3. The Adsorption Thermodynamics of the Poplar Leaves-Based ACs

**Figure 7:**
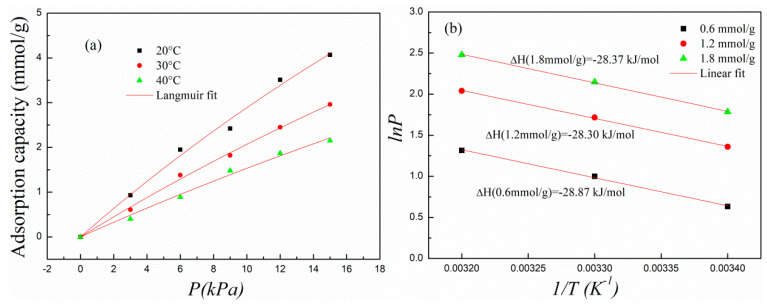
(**a**) Nonlinear fitting of the experimental adsorption data of PH0.25N1 with Langmuir model and (**b**) linear fitting with Clausius–Clapyron equation.

In view of the high adsorption capacity, PH0.25N1 was selected to further investigate the adsorption thermodynamic characteristics. The adsorption temperature and CO_2_ partial pressure were set as 20–40 °C and 0–15 kPa, respectively. The Langmuir adsorption isotherm equation was used to fit the experimental adsorption data, and the Clausius–Clapyron equation was used to calculate the isosteric heat of adsorption. Corresponding fittings are shown in Figure 7a,b, and the fitting parameters are shown in Table 2. As shown in Figure 7a and Table 2, the experimental data showed good fitting with the Langmuir equation, in which R^2^ ranged above 0.99, showing that the Langmuir model could describe the adsorption characteristic of PH0.25N1. In addition, the experimental data suggested good linear fitting with the Clausius–Clapyron equation, and the calculated isosteric heat of adsorption was almost 28 kJ/mol when the adsorption capacity ranged from 0.6 to 1.8 mmol/g, which is an indication that the adsorption of PH0.25N1 was mainly physisorption. As the adsorption temperature increased from 20 to 40 °C, the value of *k_L_* gradually decreased from 0.01316 to 0.00874, which is also a physisorption characteristic.

#### 2.2.4. Linear Correlation Analysis between the Adsorption Capacity and Influencing Factors

**Figure 8:**
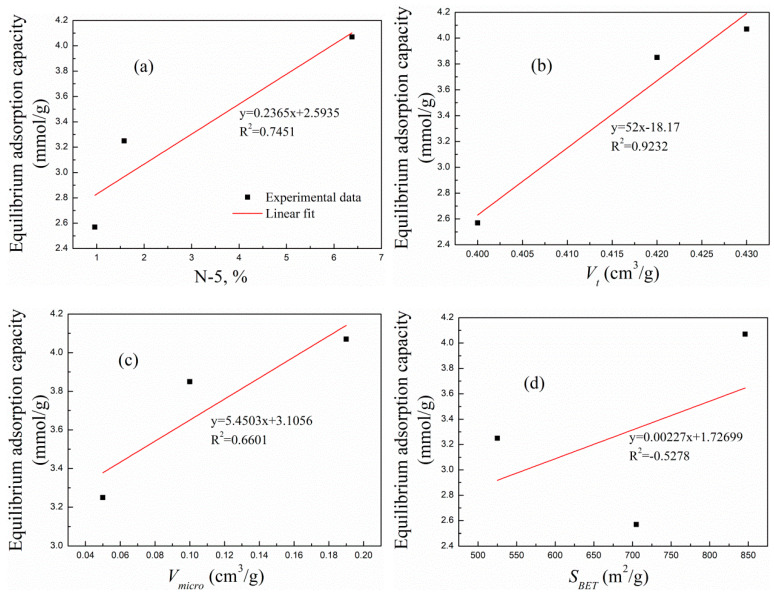
Linear fitting of the equilibrium adsorption capacity of the prepared ACs with relevant parameters. (**a**) The ratio of N-5 functional groups, (**b**) V_t_, (**c**) V_micro_, and (**d**) S_BET_.

The surface functional groups, specific surface areas, and total pore volume, especially the micropore volume, largely determine the adsorption performance of adsorbents [51]. In view of the good adsorption performance of PH0.25, PH0.25N1, PK1, and PK1N1, they were selected to study the dependence of adsorption capacity on the influencing factors, and the linear correlation fittings are shown in Figure 8. The fitting Figures indicate that the adsorption capacity did not show a good but rather a relatively weak correlation with the content of the N-5 groups, the total pore volume, the micropore volume, and the specific surface area, suggesting that none of the above factors played a decisive role in the adsorption of the poplar leaf-based ACs, but rather had a comprehensive impact.

### 2.3. The Regeneration Performance of the Selected ACs

**Figure 9:**
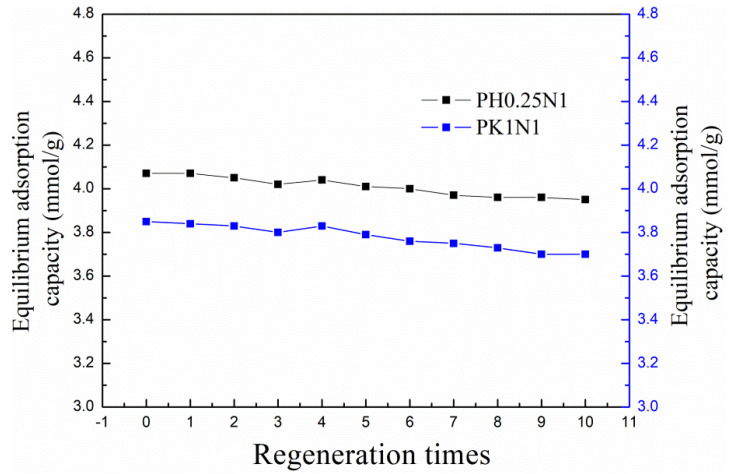
The equilibrium adsorption capacities of PH0.25N1 and PK1N1 after regeneration.

In view of their good adsorption performance, PH0.25N1 and PK1N1 were selected for further investigation of their regeneration performance. The adsorption temperature was 20 °C and the regeneration temperature was set to 200 °C according to the CO_2_-TPD results. The adsorption capacities after every regeneration for both samples are exhibited in Figure 9. As shown in Figure 9, the adsorption capacities of PH0.25N1 and PK1N1exhibited slight decreases during the ten regeneration cycles–3.95 and 3.70 mmol/g after the tenth regeneration, and were reduced by only 2.95 and 3.90%, respectively, compared with that of the fresh samples. The regeneration performance of the poplar leaf-based ACs was comparable to that reported in the related literature [30,33,50,51].

Table 3 describes the adsorption and regeneration performance of biomass-based ACs both in the literature and this work. As shown in Table 3, both the adsorption and regeneration performance of the poplar leaf-based ACs were comparable with those in the related work.

## 3. Materials and Methods

### 3.1. Materials

Poplar leaves were collected from the campus of Weifang University in Shandong, China. H_3_PO_4_ (AR, 85%) was purchased from Tianjin Kemio Chemical Reagent Co., Ltd., in Tianjin, China. KOH (GR, 85%) and urea (AR, 99%) were purchased from Shanghai Aladdin Biochemical Technology Co., Ltd., in Shanghai, China.

N_2_ (99.999%) and the simulated flue gas (85 vol.% N_2_ + 15 vol.% CO_2_) were obtained from Weiyang Gas Co., Ltd., in Yantai, China.

### 3.2. Preparation of Poplar Leaf-Based ACs

Poplar leaves were first washed with tap water and subsequently with distilled water, and then were subsequently dried in a heating oven at 80 °C. The leaves were subsequently ground into powder and sieved to less than 80 mesh for backup. Flaky KOH was also ground into powder for use.

#### 3.2.1. H_3_PO_4_– and KOH–Activated Poplar Leaves

A certain volume of H_3_PO_4_ (10.5, 21, 31.5, 42 mL) was slowly poured into a beaker containing a certain volume of dried poplar leaves (20 g, 42 mL), during which continuous stirring with a glass rod was used to process the leaves evenly soaked in H_3_PO_4_, after which the beaker was left to stand for 6 h. Subsequently, the mixture was dried at 120 °C, calcined in a muffle furnace for 1 h at 450 °C under an Ar atmosphere, washed with distilled water, and dried at 100 °C. The obtained black powder is denoted as PHa, in which P and H are poplar leaves and H_3_PO_4_, respectively, and a is the volume ratio of H_3_PO_4_ to poplar leaves. The final weight of PHa was 9.02–10.16 g, and the yield was 45.1–50.8%.

In the same way, KOH–activated poplar leaves were also prepared. However, KOH and P were mixed according to a mass ratio rather than a volume ratio, and the calcination temperature was set to 700 °C. The obtained black powder was denoted as PKc, in which K was KOH and c was the mass ratio of KOH to P, with the value of c ranging from 0.5 to 1.5. The yield of PKc was 40.7–46.2%.

#### 3.2.2. N-doping PHa and PKc

A certain mass ratio of urea to PHa or PKc was uniformly mixed and then calcined in a muffle furnace for 2 h at 350 °C under an Ar atmosphere. The mixture was subsequently washed with distilled water and dried at 100 °C. The obtained black powders were tracked as PHaNb and PKcNd, where N represents urea and b and d are the mass ratios of urea to PHa and PKc, respectively, with the value ranging from 0.5 to 1.5. The yield (the weight of the product divide by the weight of PHa or PHc) of PHaNb and PKcNd were 80–95%.

### 3.3. Characterization

The pore structures of the poplar leaf-based ACs were characterized on an ASAP 2460 (Micromeritics, Irvine, CA, USA) by the physical adsorption of N_2_ at a critical temperature of −196 °C. The BET specific surface area of S_BET_ was obtained according to the Brunauer–Emmett–Teller (BET) equation, the total pore volume V_t_ was calculated from the N_2_ adsorption amount as the relative pressure of P/P_0_ was 0.996, the micropore volume V_micro_ was obtained according to the t-plot curves, and the pore size distribution was obtained from the desorption branch according to the density functional theory method and BJH method.

X-ray photoelectron spectroscopy (XPS) characterization was performed on an EscaLab 250Xi (Thermo Scientific, Waltham, MA, USA), and the functional group ratios of N and O species were determined according to the XPS spectra.

Scanning electron microscopy (SEM) characterization of the poplar leaf-based ACs was performed on a JSM-7500F scanning electron microscope (JEOL, Showa City, Tokyo, Japan) at 5.0 kV, and surface morphology images were collected.

CO_2_ temperature-programmed desorption (TPD) characterization was also performed on a TP-5080 (Xianquan, Tianjin, China). The heating rate was 1 °C/min, and the maximum temperature was 200 °C.

### 3.4. Adsorption and Regeneration

CO_2_ adsorption and regeneration experiments were conducted on a self-assembled fixed-bed reactor. The inner diameter of the quartz loading tube is 0.8 cm, and the wall thickness is 0.1 cm. The gas chromatograph was connected at the outlet of the reactor, and the CO_2_ concentration was recorded. Firstly, 0.4 g of the ACs was laid flat in a quartz tube, N_2_ was introduced to purge the adsorbed CO_2_ and H_2_O, etc., and the reactor was adjusted to the predetermined adsorption temperature. Here, N_2_ was transferred to the simulated flue gas, after which the adsorption process began. C_0_ and C represent the CO_2_ concentrations at the inlet and outlet of the reactor, respectively. As adsorption proceeded, C gradually increased to C_0_, which indicated that the CO_2_ adsorption process reached equilibrium. The inlet gas was then retransferred to N_2_, and the reactor temperature was adjusted to 200 °C to regenerate the saturated sample. When the outlet concentration of C gradually decreased to 0, the sorbent was completely regenerated, and the regeneration process was complete.

## 4. Conclusions

Fluttered poplar leaves were used to prepare ACs with abundant pore structures and N- and O-containing functional groups using H_3_PO_4_ or KOH activation and nitrogen doping methods for CO_2_ capture. When the volume ratio of H_3_PO_4_ to poplar leaves was 0.25:1 and the activation temperature was 450 °C, and the mass ratio of urea to H_3_PO_4_–activated poplar leaves was 1:1 and the calcination temperature was 350 °C, the equilibrium CO_2_ adsorption capacity for PH0.25N1 was 4.07 mmol/g at 20 °C. Similarly, when the mass ratio of KOH to poplar leaves was 1:1 and the activation temperature was 700 °C, and the mass ratio of urea to PK1 was 1:1 and the calcination temperature was 350 °C, the CO_2_ adsorption capacity for PK1N1 was 3.85 mmol/g at 20 °C. The CO_2_-TPD characterization, adsorption kinetics, and linear correlation results showed that the adsorption process involved both physisorption and chemisorption, and the content of the N-5 groups, the total and the micro pore volume, and the specific surface area had comprehensive impacts on the CO_2_ adsorption of the poplar leaf-based ACs. The calculated isosteric heat of adsorption was almost 28 kJ/mol when the adsorption capacity ranged from 0.6 to 1.8 mmol/g, which is an indication that the adsorption of PH0.25N1 was mainly physisorption; the equilibrium adsorption stage was the reta-controlling step. In addition, the selected ACs PH0.25N1 and PK1N1 showed good regeneration performance, with adsorption capacities reducing by 2.95 and 3.90%, respectively, after ten regenerations. By comprehensively considering the material cost, corrosiveness, process energy consumption and adsorption performance, H_3_PO_4_ has more advantages than KOH as an activator of poplar leaves.

As waste biomass, poplar leaves are utilized as a source raw material for preparing ACs for CO_2_ capture, and the preparation process involves low energy consumption. The prepared poplar leaf-based ACs are excellent CO_2_ sorbents with potential applications.

## Figures and Tables

**Table 1:** Nonlinear fitting parameters of the experimental adsorption data of H_3_PO_4_– or KOH–activated poplar leaves before and after N-doping according to different kinetic models.

Kinetic Model	Parameter	PN0.25	PN0.25N1	PK1	PK1N1
Pseudo -first-order	$q_{e}$ (mmol/g)	2.82	4.74	3.94	4.69
	$k_{1}$ (1/min)	0.2741	0.1610	0.1872	0.1572
	R^2^	0.9854	0.9789	0.9821	0.9802
Pseudo -second-order	$q_{e}$ (mmol/g)	3.83	6.78	5.83	6.97
	$k_{2}$ (g/mmol min)	0.0602	0.0182	0.0233	0.0163
	R^2^	0.9744	0.9704	0.9759	0.9739
Avrami	$q_{e}$ (mmol/g)	2.60	4.15	3.36	3.96
	$k_{a}$ (1/min)	0.3146	0.2016	0.2485	0.2108
	$n_{a}$	1.4272	1.5571	1.5093	1.5410
	R^2^	0.9982	0.9955	0.9955	0.9948

**Table 2:** Nonlinear fitting parameters of PH0.25N1 according to the Langmuir model.

Parameter	20 °C	30 °C	40 °C
The fitting equilibrium adsorption capacity (mmol/g)	24.84	21.23	19.07
*k* *_L_*	0.01316	0.0108	0.00874
*R* ^2^	0.9918	0.9970	0.9908

**Table 3:** The equilibrium adsorption capacity (*q_e_*) of the fresh and regenerated biomass-based ACs.

Sorbent	*q_e_* (mmol/g) of Fresh Sample	*q_e_* (mmol/g) of Sample after Ten Regenerations	Condition	Reference
SCK-800-1	1.05	0.91	25 °C, 18% CO_2_ + 82% N_2_	[37]
CK0.3-700(1)	3.49	3.44	20 °C, 15% CO_2_ + 85% N_2_	[50]
CN1K0.3-700(1)	4.58	4.52	20 °C, 15% CO_2_ + 85% N_2_	[50]
PAC-0.5K2CO3-750-0.5	2.41	2.38	20 °C, 15% CO_2_ + 85% N_2_	[51]
PK1N1	3.85	3.70	20 °C, 15% CO_2_ + 85% N_2_	This work
PH0.25N1	4.07	3.95	20 °C, 15% CO_2_ + 85% N_2_	This work

## Data Availability

The data that support the findings of this study are available from the corresponding author, X. Wang, upon reasonable request.

## Supplementary Materials

The following supporting information can be downloaded at: https://www.mdpi.com/article/10.3390/molecules29092024/s1. Figure S1: The prediction of the rate-controlling step of PH0.25N1 by the intraparticle diffusion model; Table S1: The textural pore properties of H_3_PO_4_– or KOH-activated poplar leaves before and after N-doping; Table S2: The peak area ratios of the O species in H_3_PO_4_– or KOH-activated poplar leaves before and after N-doping; Table S3: The peak area ratios of the N species in H_3_PO_4_– or KOH-activated poplar leaves before and after N-doping.
