# Supplementary material for: The Resource Utilization of Poplar Leaves for CO2 Adsorption
Source: Molecules. 2024 Apr 27;29(9):2024. doi: 10.3390/molecules29092024 (PMC11085795; doi:10.3390/molecules29092024)
Supplement: Supplementary file 1 [file molecules-29-02024-s001.zip › molecules-2938809-supplementary.pdf]

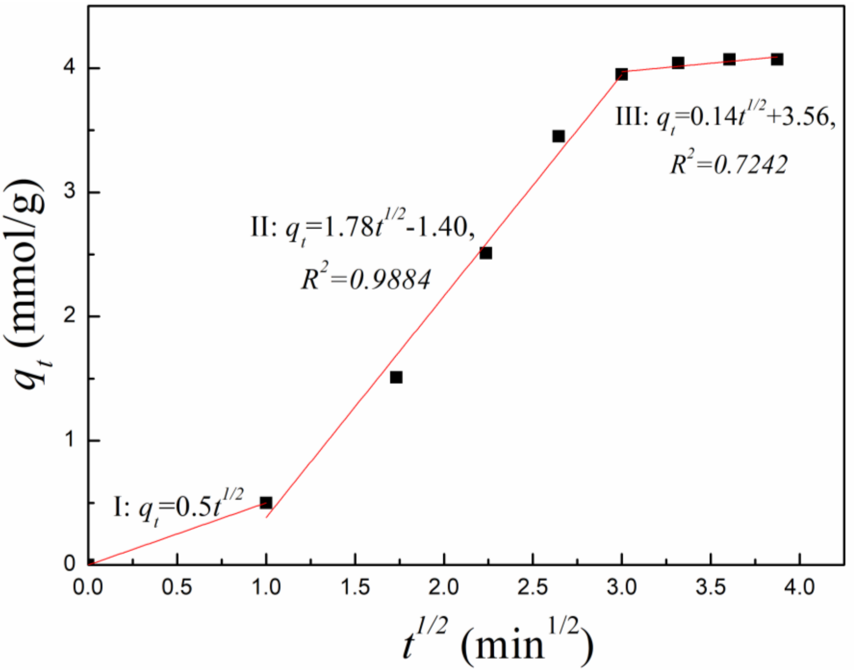

Figure S1. The prediction of the rate-controlling step of PH0.25N1 by the intraparticle diffusion model.

Table S1. The textural pore properties of H_3PO_4- or KOH-activated poplar leaves before and after N- doping.

Sample	S_{BET} (m^2/g)	V_t (cm^3/g)	V_{micro} (cm^3/g)	Micro-porosity (%)	Average pore diameter (nm)
PH0.25	705	0.40	0.08	20	2.26
PH0.25N0.5	635	0.32	0.12	37.5	2.02
PH0.25N1	846	0.43	0.19	44.2	2.02
PH0.25N1.5	720	0.36	0.11	30.6	2.00
PK0.5	499	0.30	0.08	26.7	2.40
PK0.5N1	257	0.16	0.05	31.2	2.52
PK1	525	0.45	0.05	11.1	3.43
PK1N1	416	0.42	0.10	23.8	4.04

Table S2. The peak area ratios of the O species in H_3PO_4- or KOH-activated poplar leaves before and after N- doping.

Sample	O at %	O species ratio, %				−OH at %	−C=O at %	C−O at %	−COOH at %
		−OH	−C=O	C−O	−COOH				
PH0.25	23.73	11.64	28.30	34.88	25.18	2.76	6.72	8.28	5.97
PH0.25N1	19.81	27.28	29.15	24.27	19.30	5.40	5.77	4.81	3.82
PK1	48.27	32.07	33.76	18.63	15.54	15.48	16.30	8.99	7.50
PK1N1	35.81	34.00	32.21	18.32	15.47	12.18	11.53	6.56	5.54

Table S3. The peak area ratios of the N species in H_3PO_4- or KOH-activated poplar leaves before and after N- doping.

Sample	N at %	N species ratio, %			N−6 at %	N−5 at %	N−Q at %
		N−6	N−5	N−Q			
PH0.25	2.21	10.47	43.30	46.23	0.23	0.96	1.02
PH0.25N1	9.88	21.90	64.59	13.51	2.16	6.38	1.33
PK1	2.61	19.94	80.05	0.01	0.52	2.09	0.00
PK1N1	3.92	41.01	40.29	18.70	1.61	1.58	0.73
